# Draft genomes of four multidrug-resistant (MDR) Gammaproteobacteria from biofilm on hemodialysis catheters in Gujarat, India

**DOI:** 10.1128/mra.00979-25

**Published:** 2026-01-28

**Authors:** Rajsekhar Adhikary, Sayan Poddar, Dhara Patel, Tanmoy Sen, Sishir Gang, Debarati Chattopadhyay, Uttam Kumar Nath, Saugata Hazra

**Affiliations:** 1 Department of Bioscience and Bioengineering, Indian Institute of Technology-Roorkee 30112, Roorkee, Uttarakhand, India; 2 Department of Medical Laboratory Technology, Bapubhai Desaibhai Patel Institute of Paramedical Sciences (BDIPS), Charotar University of Science and Technology, CHARUSAT Campus, Changa 130172, Anand, Gujarat, India; 3 Department of Nephrology, Muljibhai Patel Urological Hospital 29025, Nadiad, Gujarat, India; 4 Department of Burns and Plastic Surgery, AIIMS Rishikesh 442339, Rishikesh, Uttarakhand, India; 5 Department of Medical Oncology and Haematology, AIIMS Rishikesh 442339, Rishikesh, Uttarakhand, India; 6 Centre for Nanotechnology, Indian Institute of Technology-Roorkee 30112, Roorkee, Uttarakhand, India; University of Pittsburgh School of Medicine, Pittsburgh, Pennsylvania, USA

**Keywords:** antimicrobial resistance, broad-spectrum antibiotic resistance gene cassette, haemodialysis catheter, biofilm, Gammaproteobacteria, whole genome sequencing

## Abstract

Four Gram-negative bacteria, *viz. Pseudomonas aeruginosa* HL_CHRF_S30, *Klebsiella quasipneumoniae* HL_CHRU_S36A, *Enterobacter bugandensis* HL_CHRU_S49, and *Acinetobacter haemolyticus* HL_CHRU_S74 were isolated from the biofilm of the catheter tip of 152 renal failure patients. Multiple antibiotic-resistant gene cassettes were predicted by whole-genome analysis of these Gammaproteobacteria to develop antibiotic resistance.

## ANNOUNCEMENT

Catheter-related bloodstream infections (CRBSIs) pose a significant clinical challenge, especially in patients with end-stage renal disease (ESRD) who frequently necessitate prolonged vascular access. These patients are more likely to get healthcare-associated infections (HAIs), which often involve multidrug-resistant (MDR) pathogens that caused an estimated 4.95 million deaths around the world in 2019 (1, 2). This study identified biofilms on the internal lumen of tunneled cuffed catheter tips from 152 renal failure outpatients up to October 2024 at Muljibhai Patel Urological Hospital, Nadiad, Gujarat, India (Institutional ethical approval No.: MPUH/EC/788/2022), containing four isolates HL_CHRF_S30, HL_CHRU_S36A, HL_CHRU_S49, and HL_CHRU_S74, resistant against broad spectrum antibiotics *viz.* penicillin, cephalosporins, carbapenems, aminoglycosides, macrolides, etc. To isolate the pathogens, the tip was disinfected with ethanol, then opened, suspended in peptone water, and incubated at 37°C overnight. The following day, bacterial colonies were streaked on nutrient agar plates in aerobic conditions (3). Stock cultures were kept at −20°C in 20% NB-glycerol for prolonged storage (3). For genomic analysis, a single colony was recultured at 37°C, and genomic DNA was extracted using phenol-chloroform method with acceptable purity (OD₂₆₀/₂₈₀ = 1.8). The 16S rRNA gene was amplified with primers 8F (5′-AGAGTTTGATCCTGGCTCAG-3′) and 1492R (5′-GGTTACCTTGTTACGACTT-3′) and sequenced using the Sanger method (4–6). Phylogenetic analysis revealed *Pseudomonas* sp. HL_CHRF_S30 (100% sequence homology with *P. aeruginosa* JCM 5962ᵀ, BAMA01000316), *Klebsiella* sp. HL_CHRU_S36A (99.86% to *K. quasipneumoniae* subsp. *quasipneumoniae* 01A030ᵀ, HG933296), *Enterobacter* sp. HL_CHRU_S49 (99.86% to *E. bugandensis* EB-247, FYBI01000003), and *Acinetobacter* sp. HL_CHRU_S74 (99.73% to *A. haemolyticus* CIP 64.3ᵀ, APQQ01000002) according to the EzBioCloud database v2025.04.21 (6).

The NEBNext Ultra II DNA Library Prep Kit was used to make paired-end sequencing libraries. The Illumina NovaSeq 6000 platform (2 × 151 bp reads; Biokart Genomic Lab, Bengaluru, India) (7) was used for sequencing. Using TrimGalore v0.6.10, adapter sequences were removed and trimmed low-quality bases to maintain the mean Phred score 38 before assembly (1). Unicycler v0.4.8 (8) made new assemblies, and NCBI Prokaryotic Genome Annotation Pipeline (PGAP) v6.10 (9) added information to the genomes. The species was identified by the PubMLST database (https://pubmlst.org/bigsdb?db=pubmlst_rmlst_seqdef) and the rST number was recorded. The identifiable plasmid replication genes in the assembly were identified using PlasmidFinder 2.1 (https://cge.food.dtu.dk/services/PlasmidFinder/). Table 1 shows the assembly characteristics and the respective resistant gene profiles.

**TABLE 1:** Genome properties and antibiotic-resistant genes of four pathogenic Gammaproteobacteria

Parameters		Isolates details			
		*Pseudomonas aeruginosa* HL_CHRF_S30 (rST-242670)	*Klebsiella quasipneumoniae* HL_CHRU_S36A (rST-200960)	*Enterobacter bugandensis* HL_CHRU_S49 (rST-194451)	*Acinetobacter haemolyticus* HL_CHRU_S74 (rST-68447)
Genome characters	Total length (bp)	6,263,663	5,639,244	4,768,488	3,414,454
	No. of reads (Million)	5.6	6.6	6.8	8.8
	GC%	66	57	55	40
	Genome coverage	97×	97.2×	97.6×	97.8×
	N_50_ length (kb)	302.2	278.1	298	80.5
	No. of contigs	81	80	40	129
	No. of Coding sequence	5,777	5,496	4,534	3,252
	No. of rRNA	3	5	4	3
	No. of tRNA	58	74	74	65
	No. of ncRNA	4	10	9	4
	No. of plasmids	0	5	6	6
Antibiotic resistance genes		*PDC-5, OXA-396, PAO*, *emrE*, *mexP, nalD, CpxR, MexJ, MexL, ArmR, MexA, OprM, ParR, MuxC, MuxB, TriA, TriC*.	*OKP-A-11, DHA-1*, *APH(3'')-Ib*, *baeR, APH (6)-Id, cpxA, CRP, KpnG, KpnF, H-NS, KpnH, LptD, oqxA, rsmA, acrA, ramA,*	*ACT-49*, *baeR, baeS, cpxA, CRP, KpnG, KpnF, KpnE, H-NS,* OmpK37	*OXA-58, OXA-264, ADC-8, NDM-1*, *AAC (3)-IId, APH(3')-VIa, mphE, msrE, adeJ, adeK, adeI, MexK, AmvA, adeN, abeM*)*,*
Data availability	Bioproject no.	PRJNA1240882			
	Biosample no.	SAMN47739825	SAMN47739916	SAMN47739920	SAMN47740128
	GenBank accession	JBMRJT000000000	JBMRJU000000000	JBMRJV000000000	JBMRJW000000000
	SRA accession	SRR32973148	SRR32973147	SRR32973146	SRR32973145

CARD v6.0.5 and ResFinder v4.7.2 (10, 11) were used to find antibiotic resistance genes. The four isolates contained various resistance determinants, including β-lactamases (*PDC-5*, *OXA-58*, *OXA-264, OXA-396, PAO, OKP-A-11, DHA-1, ACT-49, ADC-8, NDM-1*), aminoglycoside resistance genes (*APH(3″)-Ib*, *AAC (3)-IId*, *APH(3′)-VIa*, *baeR*, *baeS*, *APH (6)-Id*, *cpxA*, *emrE*), and macrolide resistance regulators and efflux components (*CpxR, mexP, nalD, MexJ, MexL, ArmR, CRP, KpnG, KpnF, KpnE, H-NS, KpnH, MexA, OprM, ParR, MuxC, MuxB, mphE, msrE, adeJ, adeK, adeI, MexK, AmvA, adeN*). Porins (OmpK37) and multidrug efflux pumps (*LptD, oqxA, TriA, TriC, rsmA, acrA, ramA, abeM*) were found for the MDR traits of these Gammaproteobacteria (Table 1). These results underscore the intricate resistome of catheter-associated biofilm-forming pathogens. Additional genome mining is essential to understand the molecular mechanisms of antibiotic resistance in these clinically significant MDR Gammaproteobacteria.

## Data Availability

The BioProject and BioSample accession numbers of the complete genome are SAMN47739825, SAMN47739916, SAMN47739920, and SAMN47740128, respectively for *Pseudomonas aeruginosa* HL_CHRF_S30, *Klebsiella quasipneumoniae* HL_CHRU_S36A, *Enterobacter bugandensis* HL_CHRU_S49, and *Acinetobacter haemolyticus* HL_CHRU_S74. The NCBI SRA accession numbers are SRR32973148, SRR32973147, SRR32973146, and SRR32973145 and the GenBank accession numbers JBMRJT000000000, JBMRJU000000000, JBMRJV000000000, and JBMRJW000000000 for *Pseudomonas aeruginosa* HL_CHRF_S30, *Klebsiella quasipneumoniae* HL_CHRU_S36A, *Enterobacter bugandensis* HL_CHRU_S49, and *Acinetobacter haemolyticus* HL_CHRU_S74, respectively.
